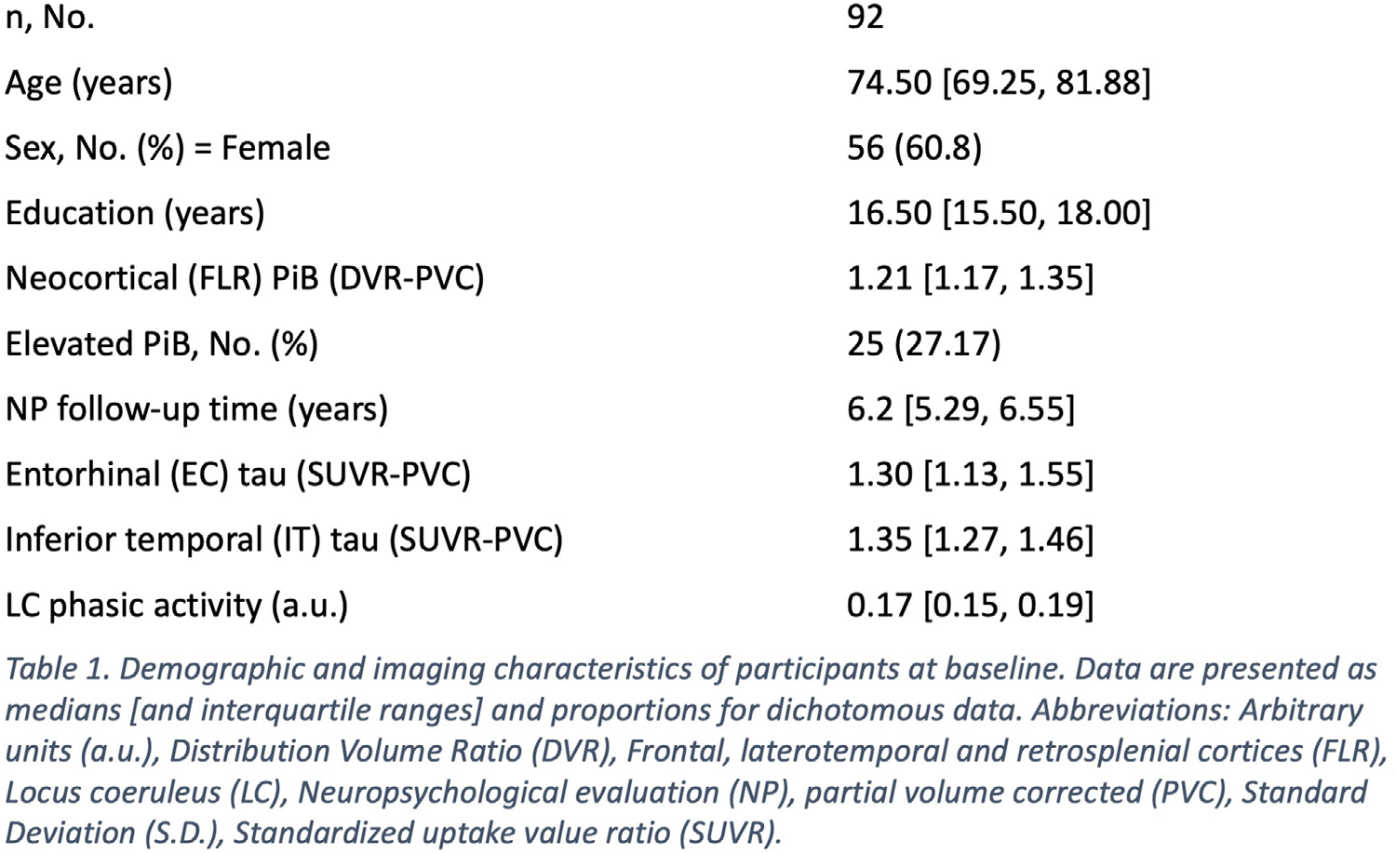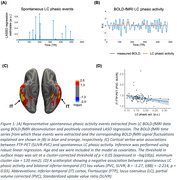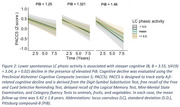# Lower resting‐state phasic LC activity is associated with cortical tau deposition and Aβ‐related cognitive decline in preclinical Alzheimer’s Disease

**DOI:** 10.1002/alz.095630

**Published:** 2025-01-09

**Authors:** Prokopis C. Prokopiou, Aaron P Schultz, Dorene M Rentz, Reisa A Sperling, Keith A Johnson, Heidi I.L. Jacobs

**Affiliations:** ^1^ Athinoula A. Martinos Center for Biomedical Imaging, Department of Radiology, Massachusetts General Hospital, Harvard Medical School, Charlestown, MA USA; ^2^ Center for Alzheimer’s Research and Treatment, Brigham and Women’s Hospital, Massachusetts General Hospital, Harvard Medical School, Boston, MA USA; ^3^ Gordon Center for Medical Imaging, Massachusetts General Hospital, Harvard Medical School, Boston, MA USA; ^4^ Faculty of Health, Medicine and Life Sciences, School for Mental Health and Neuroscience, Alzheimer Centre Limburg, Maastricht University, Maastricht, Limburg Netherlands

## Abstract

**Background:**

The locus coeruleus (LC) is one of the earliest regions accumulating tau pathology in Alzheimer’s Disease (AD). As the disease progresses, tau in the LC has been linked to increasing cortical tau and amyloid‐beta (Aβ) pathologies and cognitive decline. Previous animal research suggested that novelty‐like phasic LC activity protects against AD‐related cognitive decline. In this work, we investigate whether in‐vivo resting‐state phasic LC activity is associated with AD pathology and cognitive decline in preclinical AD.

**Method:**

Ninety‐two participants (56 Female, mean age at baseline = 74.99±9.0 years) from the Harvard Aging Brain Study underwent longitudinal cognitive testing (mean follow‐up = 5.37±1.88 years), PiB(Aβ)‐PET, FTP(tau)‐PET, and 3T resting‐state BOLD‐fMRI scans (TR/TE = 800/37 ms, voxel = 2 mm^3^) performed within 1.5 years of each other (mean time difference = 0.96±0.0 years; Table 1). Spontaneous phasic LC activity events were extracted directly from the LC BOLD‐fMRI time‐series using deconvolution and positively‐constrained LASSO regression. The proportion of LC activity explained by the detected events was quantified using the R‐squared coefficient of determination. Vertex‐wise linear regression analyses adjusted for age, sex, and multiple comparisons (p_cl_<0.05) were used to detect significant associations between LC phasic activity and cortical tau. Longitudinal linear mixed‐model analyses adjusted for age, sex, and years of education were used to evaluate the relationship between LC phasic activity and cognitive decline (composite, see Fig. 2 caption) at different levels of cortical Aβ.

**Result:**

Representative spontaneous LC phasic events and their associated BOLD‐fMRI signal fluctuations are illustrated in Fig. 1A‐B, respectively. Lower resting‐state phasic LC activity was associated with greater bilateral inferior‐temporal tau deposition (Fig. 1C), also confirmed by a region of interest analysis (Fig. 1D; *p =* 0.03). No relationship between phasic LC activity and Aβ was observed. Furthermore, lower LC phasic activity was associated with steeper cognitive (Fig. 2; *p* = 0.02) decline, particularly at elevated Aβ levels.

**Conclusion:**

This preliminary work demonstrates that lower levels of spontaneous phasic LC activity are related to elevated cortical tau pathology and steeper downstream AD‐related cognitive decline. These results could inform the design of targeted interventions to support optimal LC phasic activity, promoting resilience. Further research is needed to elucidate these relationships further by utilizing longitudinal data on AD pathology and cognitive function across different subdomains.